# Daily probiotic supplementation on plaque pH and gingival health in adults: A clinical study

**DOI:** 10.6026/973206300212365

**Published:** 2025-08-31

**Authors:** Pranav V. Manek, Alfiya M., Rashmi Sinha, Onteru Pradeep, Heena Dixit, Deepak Sharma, Rahul Tiwari

**Affiliations:** 1 Department of Oral Medicine and Radiology, Pacific Dental College and Research Centre, Udaipur, Rajasthan, India, Boston University, GMS, Chobanian & Avedisian School of Medicine, Boston, MA, USA; 2 Department of Public Health Dentistry, Yenepoya Dental College, Mangalore, Karnataka, India; 3 Department of Dentistry, Nalanda Medical College Hospital, Agamkuan, Patna, Bihar, India; 4 Department of Public Health Dentistry, Father Colombo Institute of Medical Sciences, Warangal, Telangana, India; 5 Department of Medical Health Administration, Index Institute, Malwanchal University, Index City, Nemawar Road, Indore, Madhya Pradesh, India; 6 MHA, IIHMR University, Jaipur, Rajasthan, India

**Keywords:** Probiotics, gingival index, plaque pH, oral microbiome, preventive dentistry

## Abstract

Oral health is strongly influenced by the balance of the oral microbiome, with plaque pH and gingival status serving as critical indicators of dental and periodontal well-being. A randomized, controlled clinical trial was conducted involving 60 healthy adults aged 20-45 years. The probiotic group exhibited a significant increase in plaque pH (from 5.89 ± 0.12 to 6.52 ± 0.10, p < 0.001) and a marked reduction in gingival index scores (from 1.83 ± 0.18 to 0.97 ± 0.15, p < 0.001). The control group showed no significant changes. Daily probiotic intake effectively enhanced plaque pH and gingival health, supporting its role as a preventive adjunct in routine dental care.

## Background:

Oral health plays a crucial role in overall systemic well-being, with the maintenance of optimal plaque pH and gingival health being essential for preventing common oral diseases such as dental caries and periodontal conditions [1]. The oral cavity harbors a complex and dynamic microbiome, with the balance between beneficial and pathogenic microorganisms greatly influencing oral health outcomes [2]. Among the multiple factors influencing microbial homeostasis, diet, oral hygiene practices and host immune responses are widely recognized. Recently, attention has turned to the potential of probiotics-live microorganisms that, when administered in adequate amounts, confer a health benefit on the host-as a novel adjunct in maintaining oral microbial balance and promoting gingival health [3, 4]. Probiotics exert their beneficial effects by competitive inhibition of pathogenic bacteria, enhancing mucosal immunity and regulating local inflammatory responses [5]. They have been particularly explored for their role in modulating pH levels in dental plaque, thus contributing to a less cariogenic and less inflammatory oral environment [6]. Regular consumption of probiotic strains, especially Lactobacillus and Bifidobacterium, has shown promising results in reducing plaque accumulation, gingival inflammation and oral malodor [7]. Furthermore, by buffering the pH and creating a more neutral oral environment, probiotics may inhibit the growth of acidogenic and aciduric bacteria, which are primary culprits in enamel demineralization and gingival irritation [8]. While several studies have examined the efficacy of probiotics in oral health, there remains a need for clinical trials focusing on daily supplementation in adults without systemic disease, particularly in real-world dental settings [9]. The outcomes may inform clinical practice on non-invasive, dietary-based strategies for oral health maintenance [10]. Therefore, it is of interest to evaluate the effectiveness of daily probiotic supplementation on plaque pH and gingival health in healthy adults over a specified intervention period.

## Materials and Methods:

This clinical research was conducted as a prospective, interventional trial over a period of 30 days. A total of 60 healthy adult participants aged between 20 and 45 years were recruited from the outpatient department of a dental teaching hospital. Inclusion criteria comprised systemically healthy individuals with a minimum of 20 natural teeth and mild to moderate gingivitis. Exclusion criteria included recent antibiotic therapy, use of probiotics or mouthwashes within the past three months, systemic illnesses, pregnancy, smoking, and orthodontic appliances. Participants were randomly divided into two equal groups (n = 30 each): Group A (Probiotic Group) received a commercially available probiotic supplement containing Lactobacillus reuteri (1 x 10^8^ CFU/day), while Group B (Control Group) received a placebo capsule identical in appearance. Both groups were instructed to maintain their regular oral hygiene routines without the use of additional antimicrobial agents. Baseline plaque pH and gingival index (Löe and Silness) were recorded on Day 0 using a microelectrode pH meter and a periodontal probe, respectively. Follow-up assessments were conducted on Day 30. Data were statistically analyzed using paired and unpaired t-tests, with p < 0.05 considered significant. Ethical approval was obtained from the institutional review board, and informed consent was taken from all participants.

## Results:

At baseline, both groups demonstrated comparable mean plaque pH levels and gingival index scores, with no statistically significant differences (p > 0.05). After 30 days of intervention, Group A (probiotic group) showed a notable increase in mean plaque pH from 5.89 ± 0.12 to 6.52 ± 0.10, while Group B (control) demonstrated a marginal change from 5.91 ± 0.14 to 5.95 ± 0.13. The increase in plaque pH in the probiotic group was statistically significant (p < 0.001), indicating a shift toward a less acidic, more neutral oral environment (Table 1 - see PDF). In terms of gingival health, Group A exhibited a significant reduction in mean gingival index score from 1.83 ± 0.18 to 0.97 ± 0.15 (p < 0.001), suggesting an improvement in gingival condition following probiotic supplementation. Group B showed a minor, statistically insignificant change in gingival index score from 1.80 ± 0.17 to 1.74 ± 0.16 (p = 0.08). These findings support the effectiveness of daily probiotic use in improving both plaque pH and gingival health (Table 2 - see PDF).

## Discussion:

The present clinical research assessed the effectiveness of daily probiotic supplementation on plaque pH and gingival health among healthy adults over a 30-day period. The findings clearly demonstrated that individuals receiving the probiotic supplement experienced a statistically significant improvement in both plaque pH and gingival index compared to those in the control group. These results highlight the growing relevance of probiotics as a supportive approach in preventive oral healthcare [11]. Probiotic therapy in dentistry operates through a multifaceted mechanism involving microbial competition, production of antimicrobial substances and modulation of the host's immune-inflammatory responses [12]. In this research, the observed increase in plaque pH from acidic to more neutral values is a critical indicator of reduced cariogenic potential. A neutral plaque environment inhibits the proliferation of aciduric pathogens such as Streptococcus mutans, which are known contributors to enamel demineralization and caries development [13]. Improvement in gingival health, as evidenced by the significant reduction in gingival index scores, may be attributed to the anti-inflammatory properties of the probiotic strain used. Probiotics are known to down regulate pro-inflammatory cytokines and reduce oxidative stress, thereby promoting periodontal tissue health [14]. This aligns with previous studies showing that Lactobacillus and Bifidobacterium strains may reduce gingival bleeding, plaque accumulation, and inflammatory markers when used consistently. On the contrary, the control group, which did not receive probiotics, showed only minimal improvement, likely attributable to routine oral hygiene maintenance. The lack of significant changes in this group supports the added value of probiotic supplementation beyond conventional care. It is noteworthy that the intervention was well-tolerated, with no reported side effects, reinforcing the safety profile of probiotic use in healthy individuals. However, several limitations must be acknowledged. The research duration was relatively short, and longer follow-up would help establish sustained benefits. Furthermore, the specific strain and dosage used may influence the outcome, highlighting the need for standardized probiotic formulations in future studies [15]. Despite high general awareness of probiotics among dental students, knowledge regarding their specific role in managing halitosis and periodontal diseases remains limited, emphasizing the need for broader clinical education [16]. Periodontitis has been associated with cytogenetic damage in peripheral leukocytes, highlighting the systemic impact of oral inflammation and the potential importance of microbiome-modulating approaches such as probiotics [17]. Overall, this clinical trial supports the hypothesis that daily intake of probiotic supplements can significantly enhance oral health by favorably modifying plaque pH and reducing gingival inflammation. The present study's findings are consistent with the meta-analysis by Gheisary *et al.* which demonstrated that probiotic supplementation significantly improves plaque control and gingival health through microbial modulation and anti-inflammatory effects [18]. As oral microbiome-based therapies continue to evolve, probiotics may emerge as an integral component of holistic dental care strategies [16, 17, 18, 19- 20].

## Conclusion:

This research demonstrated that daily probiotic supplementation over 30 days resulted in a significant improvement in plaque pH and gingival health in healthy adults. The probiotic group showed both a shift toward a more neutral oral environment and a substantial reduction in gingival inflammation, whereas the control group had negligible changes. These findings suggest that probiotics may serve as a beneficial adjunct to routine oral hygiene practices.
